# Extracorporeal Membrane Oxygenation in the Management of Tumor Lysis Syndrome in Children: A Review of Cases

**DOI:** 10.3390/jcm14082771

**Published:** 2025-04-17

**Authors:** Zere Aidynbek, Erken Kakenov, Olga Mironova, Karlygash Ydyrysheva, Tatyana Li, Vitaliy Sazonov

**Affiliations:** 1 Department of Medicine, School of Medicine, Nazarbayev University, Kerey Zhanibek Handar Street 5/1, Astana Z05K4F4, Kazakhstan; zere.aidynbek@nu.edu.kz; 2 Pediatric Anesthesiology and Intensive Care Unit, Mother and Child Health Center, University Medical Center, Turan 32, Astana Z05G9F0, Kazakhstan; 3 Department of Anesthesia and Intensive Care, Heart Center, University Medical Center, Turan 38, Astana Z05G9F9, Kazakhstan; 4 Department of Surgery, School of Medicine, Nazarbayev University, Kerey Zhanibek Handar Street 5/1, Astana Z05K4F4, Kazakhstan

**Keywords:** ECMO, oncology, pediatric, tumor lysis syndrome, critical care

## Abstract

**Background/Objectives**: Tumor lysis syndrome (TLS) is a life-threatening oncologic emergency that occurs in pediatric patients undergoing chemotherapy. Severe complications, including acute respiratory distress syndrome (ARDS), acute kidney injury (AKI), and cardiogenic shock, may require extracorporeal membrane oxygenation (ECMO) support. **Methods**: This paper is a nonsystematic review of cases that synthesizes available case reports to evaluate the efficacy and outcomes of ECMO in pediatric TLS. **Results**: A systematic search identified five cases in which ECMO was used with a mean duration of 14 days. Survival rates were favorable and ECMO played a critical role in bridging these patients through multi-organ failure. **Conclusions**: While ECMO is a viable rescue therapy for severe TLS, associated complications, such as infections, bleeding, and neurological impairment, warrant careful patient selection and management. Future studies should explore standardized guidelines for the use of ECMO in pediatric oncology patients.

## 1. Introduction

Cancer remains a leading cause of morbidity and mortality in children worldwide [1]. Advances in pediatric oncology over the past several decades have significantly improved survival rates, primarily due to the development of intensive chemotherapy regimens, targeted therapies, immunotherapy, and hematopoietic stem cell transplantation [2]. However, aggressive therapeutic interventions carry the risk of serious complications, including tumor lysis syndrome (TLS), a potentially fatal oncologic emergency [3]. According to studies, TLS can occur in pediatric hematological malignancies at a prevalence of 4.4% to 53.6%, and fatality can reach 21.4% [4]. While TLS is classically therapy-induced, it can also occur spontaneously, particularly in aggressive malignancies with high proliferative rates and tumor burden. Spontaneous TLS is less predictable and may occur prior to the initiation of cytotoxic therapy, posing a diagnostic challenge and emphasizing the need for baseline metabolic and laboratory vigilance in at-risk patients [5].

TLS is characterized by the massive release of intracellular contents into the bloodstream, resulting in characteristic metabolic derangements, such as hyperuricemia from nucleic acid degradation, hyperkalemia from intracellular potassium release, hyperphosphatemia, and secondary hypocalcemia from phosphate-binding calcium [4]. These metabolic abnormalities disrupt cellular homeostasis and can lead to acute organ dysfunction. The kidneys are most commonly affected due to the precipitation of uric acid and calcium phosphate crystals in the renal tubules, which can lead to acute kidney injury (AKI) and anuria [6]. In addition, the accumulation of potassium and phosphate can precipitate life-threatening cardiac arrhythmias, while hypocalcemia can contribute to neuromuscular irritability and seizures [7]. In severe cases, TLS can rapidly progress to multi-organ failure, affecting not only the renal and cardiovascular systems but also the central nervous system [8]. To provide a standardized framework for diagnosis, the Cairo–Bishop criteria classify TLS into two categories: laboratory TLS, defined by specific changes in serum uric acid, potassium, phosphate, and calcium levels, and clinical TLS, which includes the development of one or more clinically significant complications such as renal failure, cardiac arrhythmias, seizures, or sudden death [9]. However, recent studies have shown that the Howard criteria may more accurately identify patients at risk for adverse outcomes associated with TLS [10]. Given the high risk of rapid clinical deterioration, prevention of TLS is a critical component of management. Prophylactic strategies focus on minimizing the metabolic consequences of tumor cell lysis [11]. This includes aggressive intravenous hydration to improve renal perfusion and promote urinary excretion of uric acid and electrolytes [12]. Pharmacologic prophylaxis includes the use of allopurinol, a xanthine oxidase inhibitor that reduces uric acid production, and rasburicase, a recombinant urate oxidase that catalyzes the conversion of uric acid to the more soluble allantoin, facilitating rapid clearance. Rasburicase is particularly recommended for high-risk patients or those with established hyperuricemia [4].

While the management of TLS focuses primarily on prevention and mitigation of metabolic derangements, in severe cases, renal replacement therapy (RRT) may be required to control hyperkalemia and metabolic acidosis [13]. Despite these interventions, a subset of TLS patients develop refractory organ dysfunction, including severe heart and lung failure, requiring advanced life support, such as extracorporeal membrane oxygenation (ECMO) [14].

ECMO is a form of extracorporeal life support (ECLS) used to provide temporary cardiac and/or respiratory support to patients with severe but potentially reversible cardiopulmonary failure [15]. ECMO has been widely used in conditions, such as neonatal and pediatric ARDS, myocarditis, and septic shock [16], but its role in pediatric oncology, particularly in TLS, is still under investigation.

When TLS leads to cardiogenic shock, ARDS, or multi-organ dysfunction, ECMO may serve as a bridge to recovery, allowing time for metabolic correction and stabilization of organ function. However, the decision to initiate ECMO in TLS patients requires careful risk–benefit assessment, as oncology patients often have a compromised immune system, increased risk of bleeding, and uncertain long-term prognosis [17]. The interplay between TLS-related metabolic disorders and ECMO-related complications further complicates the management of these patients.

This review aims to explore the published disparate cases on the use of ECMO in pediatric TLS patients.

## 2. Materials and Methods

**Figure 1:**
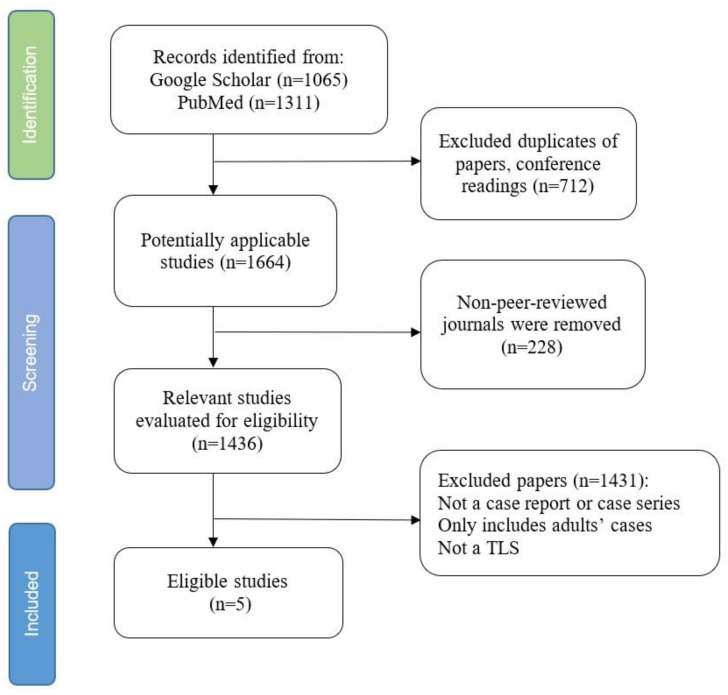
Literature screening flowchart limited to case reports and case series relevant to ECMO use in pediatric TLS.

A literature search was performed using PubMed, Google Scholar, and Scopus-indexed journals with the keywords “tumor lysis syndrome”, “extracorporeal membrane oxygenation”, and “pediatric”. Inclusion criteria were English-language case reports, case series, and retrospective studies describing the use of ECMO in TLS patients aged 0–18 years. Our search strategy reflected in Figure 1.

A total of 2376 publications were initially identified, 2076 in Google Scholar and 300 in Pubmed. Among them, 712 papers were determined to be duplications, and 228 papers were abstracts or conference papers and were thus excluded. Of the relevant papers, 1436 were evaluated for eligible children’s case reports or case series. We excluded 1431 publications for not being a case report or case series, not children’s cases, or not having a TLS diagnosis. Five case reports met all inclusion criteria after applying exclusion criteria for duplicates, conference abstracts, and non-relevant studies.

## 3. Results

The five pediatric cases (summarized in Table 1) identified involved TLS secondary to hematologic malignancies, including leukemia, B-cell lymphoma, rhabdomyosarcoma, and juvenile myelomonocytic leukemia (JMML). ECMO was initiated for respiratory failure, ARDS, cardiogenic shock, or cardiac arrest. The ECMO modality used included veno-venous (VV) ECMO for ARDS and veno-arterial (VA) ECMO for cardiac dysfunction. Some patients required conversion between VV and VA ECMO due to evolving clinical conditions. The median duration of ECMO was 14 days. Despite severe disease progression, all patients survived ECMO therapy, although some experienced long-term neurological sequelae.

## 4. Discussion

In this study, we analyzed a small group of pediatric patients with TLS who required ECMO support due to severe respiratory or cardiac failure. Given the rarity of ECMO use in TLS, our review provides insights into clinical indications, management strategies, and patient outcomes. In this section, we discuss key findings, challenges in the use of ECMO in TLS, and considerations for future clinical practice.

TLS is known to typically manifest within 48–72 h (2–3 days) after the start of chemotherapy [23]. However, in some cases, particularly in highly proliferative malignancies, it can occur within a few hours. The cases reviewed in this study confirm this concept: the earliest onset of TLS occurred within the first 24 h (case one in Table 1), while the latest onset was observed on day 4 (the fourth case). This underscores the importance of continuous monitoring for TLS beyond the initial 48-h period. TLS is most commonly associated with hematologic malignancies, particularly acute leukemias and high-grade lymphomas, due to their high cell turnover rates and sensitivity to chemotherapy [24]. However, TLS can also occur in solid tumors, particularly those with a high tumor burden or high proliferative index [25]. Although TLS does not develop in all pediatric oncology patients, clinicians should remain vigilant, as early recognition and intervention can significantly impact outcomes.

The use of ECMO in pediatric TLS patients presents both opportunities and challenges. While ECMO has been lifesaving in cases of severe respiratory or cardiac failure, its use in oncologic emergencies remains controversial due to concerns about prognosis, immunosuppression-related complications, and long-term outcomes [26].

A key consideration in ECMO management is the selection of the appropriate modality. The two main types of ECMO are veno-arterial (VA) ECMO, which provides both cardiac and respiratory support, and veno-venous (VV) ECMO, which provides respiratory support only [27]. In this review, VA-ECMO was predominantly used in the setting of cardiogenic shock, whereas VV-ECMO was the preferred modality in patients with isolated respiratory failure. The conversion from VA to VV-ECMO observed in some cases suggests that initial hemodynamic instability may improve with metabolic correction and time, allowing a transition to less invasive support. For example, the 8-year-old boy (case number three) was initially put on VV ECMO for acute pulmonary edema. However, several cardiac arrests took place and the risk of pulmonary hypertension led to switching from VV to VA ECMO [20]. Similarly, a 4-year-old boy (case two) with ARDS and cardiogenic shock required immediate VA-ECMO. Improvements in ventilation were observed on day 12 on ECMO support and on day 18, VA ECMO was converted to VV ECMO [19]. The last case (number five) involves a 16-month-old boy supported by VA ECMO. After 16 days, VA was converted to VV ECMO [22]. These findings underscore the importance of careful patient monitoring and dynamic adaptation of the ECMO strategy during the course of treatment [28].

Beyond modality selection, another critical consideration in ECMO management is effective monitoring. Given the complex interplay between ECMO support, systemic inflammation, and metabolic derangements, pediatric patients remain vulnerable to brain injury. That is why continuous neurological monitoring during ECMO is essential to detect early signs of brain injury, guide sedation and anticoagulation management, and optimize perfusion strategies to minimize neurological sequelae [29]. The case (number two) reported by Bartkevics et al. [19] is an initial account of a child’s case of JMML and ARDS triggered by TLS that survived a long term ECMO administration for 25 days. The 4-year-old was put on ECMO for signs of several TLS complications, like pulmonary infiltration, pulmonary hypertension, and lactic acidosis on top of ARDS. Even though his breathing was stabilized after ECMO, the child developed quadriplegia, global aphasia, conjugate eye deviation, and a bilateral vocal cord paralysis. The neurological deficits may be due to prolonged ECMO duration, metabolic derangements, or hypoxic events prior to ECMO initiation. Even though this patient still had mild cognitive damage at the time of discharge, his life was saved with the help of an ECMO machine. Wang et al. (2022) [18], in their case (number one), also emphasized the need for neuro-monitoring, which can be done with the bispectral index (BIS) or cerebral blood flow signals monitored by transcranial doppler, or even a combination of these methods.

Continuous and comprehensive laboratory monitoring is essential to detect early metabolic changes and guide timely intervention. Key parameters to monitor include serum uric acid, potassium, phosphate, calcium, creatinine and lactate dehydrogenase (LDH), with frequent reassessments during the early days of therapy. Early identification of laboratory abnormalities through vigilant monitoring allows clinicians to escalate preventive or therapeutic interventions and prevent progression to clinical TLS [30].

Another critical aspect of ECMO in TLS patients is the potential for complications. While the exact prevalence of complications in this population remains unclear, hemorrhagic events have been reported as a common problem, often requiring anticoagulation adjustments to mitigate the risk of bleeding [22]. Infections were also a major concern, particularly in immunocompromised patients undergoing chemotherapy [18]. As ECMO involves the use of intravenous catheters, it significantly increases the incidence of patients getting catheter-related bloodstream infections (CRBSIs) [31]. Healthcare-related infections, like CRBSIs, cause a rise in mortality of 38–63% in ECMO patients [32]. When a child is on ECMO assistance for more than five days, the likelihood of CRBSIs gets higher [33]. Antibiotic therapy was intensified during the ECMO connection phase in all cases included in our study. Prophylactic antibiotic strategies and strict infection control protocols should be incorporated into the management of ECMO patients with TLS to optimize outcomes.

In addition to antibiotic therapy, especially if patients still indicate elevated signs of inflammation, intravenous immunoglobulin can be provided to strengthen their immune system. Despite having various protective measures, being on ECMO for a short period of time is the best way to stay away from nosocomial infections [34]. At the same time, when pediatric cancer is combined with sepsis, ECMO allows additional extracorporeal blood purification methods to be added to the circuit. Cases have been reported where hemosorption in cancer patients has significantly improved the outcome of the underlying disease [35,36].

Renal dysfunction was another common problem, with many patients requiring continuous renal replacement therapy (CRRT). The patients who exhibit dehydration or hypotension (due to shock for example) are at greater risk of developing acute kidney injury (AKI) due to their lower renal perfusion [13]. Integration of CRRT with ECMO was required in four cases and was effective in stabilizing metabolic derangements and improving overall patient recovery. Indications for the initiation of CRRT in reviewed cases include oliguria or anuria, refractory hyperkalemia, persistent metabolic acidosis, and rising serum creatinine indicative of AKI. In the context of ECMO, CRRT is also used to manage fluid overload, correct severe electrolyte disturbances, and treat profound uremia.

In addition to what has already been discussed, it should be noted that ECMO has been recognized as a potentially life-saving intervention for pediatric oncology patients in several other emergency situations [37]. In certain cases, ECMO serves not only as an acute support but also as a bridge to definitive treatment. This is especially true for patients awaiting organ recovery, disease stabilization, or further oncologic intervention. Chemotherapy-induced immunosuppression and bone marrow hematopoietic failure significantly increase the risk of serious infections [14]. And ECMO has been used in children undergoing chemotherapy who develop severe infections and subsequent organ failure. An et al. [38] reported a case series of eleven pediatric patients with ARDS due to infections such as pneumocystis pneumonia (PCP), parainfluenza, and pulmonary hemorrhage, all requiring ECMO. Of these, six had hematologic malignancies and five had solid tumors. Patients were on ECMO for an average of 22 days, primarily in the VV mode. Similarly, Gow et al. [39] analyzed 107 oncology patients who required ECMO for respiratory or cardiac failure after chemotherapy. Of these, 73 had hematologic malignancies and 34 had solid tumors. The median duration of ECMO was 6.1 days, and 37 patients survived to hospital discharge. Mortality was attributed to organ failure, bleeding, family decision to withdraw care, and prolonged critical illness. Meister et al. [14] reviewed four leukemia patients with ARDS due to infection prior to ECMO initiation. Two patients with Streptococcus oralis sepsis and zygomycosis were successfully weaned from ECMO after receiving transfusions and VV ECMO support. In contrast, two patients with multiple infections, including E. coli sepsis, pneumonia, and cerebral lesions, suffered fatal complications such as pulmonary hemorrhage and diffuse alveolar damage. All of these studies reported significantly higher mortality in children with malignancies than in typical PICU patients when ECMO was used. Although these findings must be interpreted with caution due to limited detailed data, they suggest that ECMO remains a viable salvage option for carefully selected patients with malignancies. Furthermore, it is important to recognize that in some cases, ECMO is the last available therapeutic intervention, underscoring the importance of careful patient selection, timely initiation, and optimal supportive care to maximize its potential benefits.

Due to lower body weight and greater susceptibility to multi-organ failure during cancer treatment, pediatric patients require highly specialized ECMO management [40]. This highlights the critical need for expertise in ECMO administration in pediatric oncology patients.

### 4.1. Clinical Implications and Decision-Making Considerations

Clinicians caring for pediatric oncology patients must remain vigilant for the potential rapid progression of TLS and associated complications. Early recognition and intervention are critical, as timely initiation of ECMO can significantly improve outcomes. Patients undergoing intensive chemotherapy, especially those with high tumor burden, should be closely monitored for early signs of severe TLS and associated organ dysfunction. Given the complexity of managing TLS in patients requiring ECMO, a multidisciplinary approach is essential [41]. Effective coordination between pediatric oncologists, intensivists, ECMO specialists, and nephrologists ensures comprehensive patient care. Institutions should consider establishing clear, evidence-based protocols that outline criteria for ECMO initiation, management strategies during ECMO support, and guidelines for weaning patients from ECMO.

Selection of appropriate candidates for ECMO support requires careful consideration of the patient’s overall prognosis, reversibility of organ dysfunction, and potential ECMO-related risks [42]. ECMO should be reserved for patients with significant but potentially reversible cardiopulmonary dysfunction. The decision-making process should include a thorough risk-benefit assessment, taking into account individual patient factors and clinical circumstances. Neurological monitoring is a critical component of ECMO management due to the potential for neurological complications, particularly with prolonged ECMO support [43]. Ongoing assessment and early intervention, including strategies for optimized perfusion and targeted temperature management, can help reduce neurological risk and improve patient outcomes.

Ultimately, the clinical decision-making process should be guided by standardized protocols and institutional readiness to facilitate consistent and evidence-based patient management. Future multicenter studies are needed to further refine these recommendations and establish robust guidelines for the use of ECMO in pediatric TLS.

### 4.2. Limitations

There are several limitations to this study that must be acknowledged. Due to the rarity of ECMO use in TLS, the available literature is primarily limited to case reports. These are inherently subject to publication bias, as successful cases are more likely to be reported, while unsuccessful or complicated cases may be underrepresented.

Another one of the inherent limitations of this type of research is the lack of standardized and detailed reporting across case studies. Of the included cases, only one was described in detail, while others provided limited clinical information—often only that certain parameters had increased or decreased, without specifying levels or trends. In most reports, available data were limited to the time of transfer to the PICU, with limited insight into disease progression or response to treatment. In addition, none of the authors used the Cairo–Bishop criteria or any other to classify TLS, and only one report specified the exact chemotherapy protocol used. This heterogeneity significantly limits our ability to make consistent comparisons and draw robust conclusions across cases.

Consequently, the results of this review may not fully reflect the broader spectrum of ECMO outcomes in pediatric TLS patients. The heterogeneity of the reported cases, including variations in malignancy types, ECMO indications, and management strategies, limits the ability to draw definitive conclusions. Differences in institutional protocols and availability of ECMO further contribute to the variability in patient outcomes. The lack of large-scale retrospective or prospective studies on the use of ECMO in pediatric TLS presents a challenge in establishing evidence-based guidelines. Future research should focus on collecting multicenter data to better understand risk factors, survival predictors, and best practices for ECMO in this population. Finally, long-term follow-up data on neurological and functional outcomes of ECMO survivors remain scarce. As some of the cases reviewed reported neurological impairment, further investigation is needed to assess the impact of ECMO on cognitive and motor function in pediatric TLS patients.

## 5. Conclusions

ECMO is a viable therapeutic option for pediatric TLS patients with severe organ failure. Although survival outcomes are promising, clinicians must carefully weigh the risks and benefits when considering ECMO in immunocompromised patients. Future multicenter studies are needed to establish evidence-based guidelines for the use of ECMO in the management of pediatric TLS.

## Figures and Tables

**Table 1:** Case reports of pediatric patients administered ECMO.

Case #	Author, Year	Age, Years, Gender	Diagnosis	Initial Chemotherapy, TLS Diagnosed	ECMO Indication	ECMO Mode	Duration (days)	CRRT Used	Outcome
1	Wang et al., 2021 [18]	9, F	B-cell lymphoma (stage IV, R4)	Methotrexate. TLS developed in the next 24 h	Cardiogenic shock, cardiac arrest	VA	3 (71h)	Yes	- Successfully weaned - Survived - Discharged from the hospital on day 31
2	Bartkevics et al., 2020 [19]	4, M	JMML	Cytarabine and rasburicase. TLS developed less than 24 h	ARDS, cardiogenic shock, cardiac arrest	VA → VV	18 for VA 7 for VV 25 in total	No	- Successfully weaned off - Survived - Sustained serious neurological damage: cognitive abilities compromised - Discharged after receiving bone marrow transplantation
3	Sanford et al., 2016 [20]	8, M	Metastatic ARMS	Vincristine and irinotecan were given on therapy day 1, and irinotecan alone on day 2. On day 3 TLS developed	ARDS, Pulmonary Edema	VV → VA	5	Yes	- Successfully weaned off - Survived
4	Huang et al., 2015 [21]	18 mo, F	AML-M4Eo	cytarabine, daunorubicin, and etoposide per the COG AAML0531 protocol. TLS developed on day 4	ARDS	VA	9	Yes	- Successfully weaned off - Survived
5	Prabhu et al., 2012 [22]	16 mo, M	AML	Etoposide, daunorubicin and cytarabine. TLS developed in the next 24 h	ARDS	VA → VV	16 for VA 3 for VV 19 in total	Yes	- Complicated with thrombocytopenia, pericardial effusion, bleeding - Successfully weaned off - Completed chemotherapy and recovered

AML—Acute myeloid leukemia, JMML—Juvenile myelomonocytic leukemia, ARMS—Alveolar Rhabdomyosarcoma, ARDS—Acute respiratory distress syndrome. F—female, M—male. CRRT—Continuous renal replacement therapy.

## Data Availability

No new data were created or analyzed in this study. Data sharing is not applicable to this article.
